# Delivery outcomes in term births after bariatric surgery: Population-based matched cohort study

**DOI:** 10.1371/journal.pmed.1002656

**Published:** 2018-09-26

**Authors:** Olof Stephansson, Kari Johansson, Jonas Söderling, Ingmar Näslund, Martin Neovius

**Affiliations:** 1 Department of Medicine, Solna, Clinical Epidemiology Unit, Karolinska Institutet, Stockholm, Sweden; 2 Division of Obstetrics and Gynaecology, Department of Women’s and Children’s Health, Karolinska Institutet, Stockholm, Sweden; 3 Department of Surgery, Faculty of Medicine and Health, Örebro University, Örebro, Sweden; University of Manchester, UNITED KINGDOM

## Abstract

**Background:**

Obesity increases the risk of adverse delivery outcomes. Whether weight loss induced by bariatric surgery influences these risks remains to be determined. The objective was to investigate the risk of adverse delivery outcomes among post-surgery women compared with women without bariatric surgery history but with similar characteristics.

**Methods and findings:**

We identified 801,443 singleton live-born term births (≥37 gestational weeks) in the Swedish Medical Birth Register between 1 January 2006 and 31 December 2013, of which 1,929 were in women with a history of bariatric surgery and a pre-surgery weight available from the Scandinavian Obesity Surgery Registry. For each post-surgery delivery, up to 5 control deliveries were matched by maternal pre-surgery BMI (early-pregnancy BMI used for controls), age, parity, smoking, education, height, country of birth, and delivery year (*N* post-surgery deliveries:matched controls = 1,431:4,476). The main outcome measures were mode of delivery, induction of labor, post-term pregnancy (≥42 + 0 gestational weeks), epidural analgesia, fetal distress, labor dystocia, peripartum infection, obstetric anal sphincter injury (perineal tear grade III–IV), and postpartum hemorrhage. Among the women with a history of bariatric surgery, the mean pre-surgery BMI was 42.6 kg/m^2^, the median surgery-to-conception interval was 1.4 years, and the mean BMI loss between surgery and early pregnancy was 13.5 kg/m^2^ (38 kg). Compared to matched control women, post-surgery women were less likely to have cesarean delivery (18.2% versus 25.0%, risk ratio [RR] 0.70, 95% CI 0.60–0.80), especially emergency cesarean (6.8% versus 15.1%, RR 0.40, 95% CI 0.31–0.51). Post-surgery women also had lower risks of instrumental delivery (5.0% versus 6.5%, RR 0.73, 95% CI 0.53–0.98), induction of labor (23.4% versus 34.0%, RR 0.68, 95% CI 0.59–0.78), post-term pregnancy (4.2% versus 10.3%, RR 0.40, 95% CI 0.30–0.53), obstetric anal sphincter injury (1.5% versus 2.9%, RR 0.46, 95% CI 0.25–0.81), and postpartum hemorrhage (4.6% versus 8.0%, RR 0.58, 95% CI 0.44–0.76). Since this study was not randomized, a limitation is the possibility of selection bias, despite our efforts using careful matching.

**Conclusions:**

Bariatric-surgery-induced weight loss was associated with lower risks for adverse delivery outcomes in term births.

## Abstract 2

Using data from the Swedish Medical Birth Register and Scandinavian Obesity Surgery Registry, Kari Johansson and colleagues assess whether bariatric surgery is associated with reduced adverse maternal delivery outcomes in term births.

## Author summary

**Why was this study done?:**

- Obesity is prevalent and is a major health problem in pregnancy and childbirth.
- Bariatric surgery induces large and sustained weight loss and is becoming more common in Sweden and other developed countries.
- Whether bariatric-surgery-induced weight loss influences delivery outcomes is not well described.

**What did the researchers do and find?:**

- We compared 1,431 term births in women with bariatric surgery history with 4,476 population control births matched on pre-surgery BMI in cases and early-pregnancy BMI in controls, as well as several other maternal characteristics.
- Women with bariatric surgery history had lower risk of instrumental delivery and cesarean delivery during labor.
- Women with bariatric surgery history, compared with controls, had substantially lower risks for post-term pregnancy, induction of labor, epidural analgesia, and delivery complications such as labor dystocia, fetal distress, peripartum infection, obstetric anal sphincter injury, and postpartum hemorrhage.

**What do these findings mean?:**

- Bariatric-surgery-induced weight loss was associated with lower risks for adverse delivery outcomes in term births.
- Although bariatric surgery appears beneficial for maternal delivery outcomes, other adverse pregnancy and infant outcomes have to be considered when counseling women on the safety of giving birth after bariatric surgery.

## Introduction

Obesity in women of childbearing age has increased in the US and many other developed countries during the last decades [1]. The proportion of US women 20–39 years of age with obesity (body mass index [BMI] ≥ 30 kg/m^2^) was 35.7% in 2015–2016, and the corresponding proportion for class III obesity (BMI ≥ 40 kg/m^2^) was 7.8% [2]. Maternal obesity is associated with adverse delivery outcomes including induction of labor, cesarean delivery, labor dystocia, fetal distress, and postpartum hemorrhage [3].

Unfortunately, effective treatment options for obesity are limited. Bariatric surgery is the only treatment to date that induces large and sustained weight loss [4,5]. Although bariatric surgery is accepted as reasonably safe, uncertainty remains regarding the risks for a subsequent pregnancy and delivery [6–8]. We have previously reported lower risks of gestational diabetes and large-for-gestational-age births as well as higher risks of small-for-gestational-age birth and preterm birth in post-surgery women compared to controls matched for pre-surgery BMI [9,10]. Hence one would expect a lower risk for delivery complications.

Three meta-analyses on bariatric surgery and delivery outcomes reported non-significant differences for cesarean delivery and postpartum hemorrhage between women with and without a history of bariatric surgery [11–13]. Studies on bariatric surgery and delivery outcomes have generally been small, used heterogeneous control groups [14–19], or lacked a comparison group accounting for pre-surgery BMI [6,15,16,18–24]. We have identified only 3 previous studies on delivery outcomes that included a control group matched on pre-surgery BMI [14,17,25], of which all included fewer than 140 post-surgery deliveries. No previous study to our knowledge has investigated obstetric anal sphincter injury (OASIS; i.e., perineal tear) in women with bariatric surgery history compared with controls.

We conducted a population-based study using data from nationwide Swedish registers on singleton live term and post-term births (i.e., gestational age ≥37 completed weeks). We compared the risk of adverse delivery outcomes including cesarean delivery, instrumental delivery, post-term pregnancy (gestational age ≥42 completed weeks), induction of labor, labor dystocia, fetal distress, peripartum infection, OASIS, and postpartum hemorrhage among deliveries to women with versus without a bariatric surgery history but with otherwise similar characteristics.

## Methods

### Setting

In Sweden, prenatal and delivery care are tax funded, and the participation rate in the prenatal care program is almost 100%. The Swedish Medical Birth Register includes information on more than 98% of all births in Sweden since 1973 [26]. Information is prospectively collected during pregnancy, delivery, and the neonatal period using standardized prenatal, obstetric, and neonatal records. This matched cohort study was approved by the regional ethics committee in Stockholm, Sweden (No. 2013/730-31), and was conducted on de-identified data. By using the unique personal identification number assigned to each Swedish resident [27], data from the Medical Birth Register was linked to the Scandinavian Obesity Surgery Registry (SOReg), and the National Patient Register.

SOReg was established nationwide in 2007 and covers approximately 98.5% of all bariatric procedures in Sweden [28]. Local data from a few hospitals were available beginning in 2004. The register includes pre-surgery and follow-up information.

The National Patient Register includes diagnostic and surgical information on hospital admissions and hospital-based outpatient care visits, coded according to the Swedish versions of the International Classification of Diseases–10th revision (ICD-10) and the Nordic Medico-Statistical Committee Classification of Surgical Procedures, both used in Sweden from 1997 and onwards [29].

### Participants

We did not work from a pre-specified analysis plan, but instead used the methods described in 2 previous studies [9,10]. We had access to 876,068 deliveries recorded in the Medical Birth Register between 1 January 2006 and 31 December 2013. We excluded births to mothers without a valid personal identification number at the time of delivery, who could not be linked to other registers for assessment of bariatric surgery status. We also excluded mothers with multiple births (since they differ regarding delivery outcomes). From the remaining 844,956 singleton deliveries with linkable mothers (S1 Fig), we excluded stillbirths (0.32%) and preterm births (<37 completed gestational weeks; 4.8%), as well as deliveries where gestational age was missing (0.04%). After these exclusions, 801,443 singleton live births at or after 37 gestational weeks remained, of which 3,105 occurred after bariatric surgery (performed between 1 January 1983 and 31 December 2013). Of these, 1,956 were performed between 1 January 2006 and 31 December 2013 and were included in SOReg. From SOReg, we recorded the bariatric surgery date, the type of procedure, and pre-surgery BMI (*N =* 1,929 had data on this variable).

We created a matched control cohort using births to women without bariatric surgery history (according to the National Patient Register and SOReg). Up to 5 control births were matched without replacement to each post-surgery birth; once a birth to a woman without bariatric surgery history was selected as a control, the same birth could not be selected again. The matching factors were maternal age (± 2 years), parity (primiparous or multiparous), pre-surgery BMI category (using early-pregnancy BMI in controls; 30 to <35, 35 to <40, 40 to <45, 45 to <50 or ≥50 kg/m^2^), early-pregnancy smoking status (non-smoker, 1–9 daily cigarettes, ≥10 daily cigarettes, or missing), educational level (<10, 10–12, or >12 years, or missing), height (<155, 155 to <165, 165 to <175, or ≥175 cm), country of birth (Nordic or non-Nordic), and delivery year (2006–2013; ±1 year).

Pre-surgery BMI was calculated from measured weight and height before surgery. Early-pregnancy BMI was calculated from measured weight and self-reported height at the first prenatal visit (median gestational week 10); self-reported smoking status was also registered at the first prenatal visit. To reduce measurement error and missingness, we used the median height from all registered pregnancies. Mother’s country of birth was retrieved from the Medical Birth Register and categorized into Nordic (Sweden, Denmark, Norway, Finland, Iceland) or non-Nordic.

### Main outcome measures

The main outcomes of the study were cesarean delivery, instrumental delivery (forceps or vacuum extraction), induction of labor, post-term pregnancy (gestational age ≥ 42 completed weeks), epidural analgesia, and delivery complications including labor dystocia, fetal distress, peripartum infection, OASIS, and postpartum hemorrhage (defined as blood loss ≥ 1,000 ml within 24 hours following delivery).

Information on mode of delivery, induction of labor, epidural analgesia, and OASIS was obtained from standardized delivery record information. Information on labor dystocia (ICD-10: O62.0, O62.1, O62.2, O62.8, O62.9, O66.9, O63.0, O63.1, O63.9), fetal distress (ICD-10: O68.9), postpartum hemorrhage (ICD-10: O72, O67.8), and peripartum infection (ICD-10: O41.1, O75.2, O75.3, O85.9, O86.0) were obtained from diagnoses at hospital discharge. Cesarean delivery was further divided into elective (before labor start) and emergency. For emergency cesarean delivery, we also investigated indication, classified into dystocia, fetal distress, or other.

### Statistical analysis

In order to characterise the relationship between BMI and the selected delivery outcomes, we used cubic splines applied to the whole study population with early-pregnancy BMI available (excluding births to women with previous bariatric surgery; remaining *N =* 729,867); we characterized the relationship between BMI as a continuous variable and the outcomes with knots placed at BMI 18.5, 22, 25, 30, 35, and 40 kg/m^2^.

The association between history of bariatric surgery (versus matched controls) and delivery outcomes was estimated by risk ratios (RRs) with 95% confidence intervals (CIs) by using modified Poisson regression models [30], conditioning on the matching set, with each set consisting of 1 post-surgery birth and up to 5 matched control births.

#### Subgroup analyses in response to peer review comments

As parity is an important determinant of several delivery outcomes, we performed a subgroup analysis by this variable, and tested for effect modification by the interaction of bariatric surgery status with parity. We also performed a subgroup analysis by surgery-to-conception interval (<1 versus ≥1 year), as some national guidelines do not recommend women to become pregnant during the first year after bariatric surgery [31].

#### Mediation analysis

We performed mediation analysis by including birth weight as a continuous variable, including the matching variables as covariates, in a multivariable analysis.

#### Sensitivity analysis

Since the primary analyses were performed on individual births, making it possible for a woman to contribute more than 1 birth, we repeated the analysis restricting the cohort to include only the first birth after bariatric surgery, to remove the potential influence of the same mother giving birth to more than 1 child. (Considering only nulliparous women also addresses this issue, as done in the subgroup analysis.).

Data were analyzed using SAS (version 9.4). Two-sided *P* values < 0.05 were considered statistically significant. No adjustment was made for multiple comparisons.

## Results

Ninety-eight percent (*N =* 1,896) of bariatric surgery procedures were gastric bypass, and 1.7% (*N =* 33) were sleeve gastrectomy, gastric banding, or biliopancreatic diversion with duodenal switch. The median surgery-to-conception interval was 1.4 years (interquartile range 0.8–2.3), and the median surgery-to-delivery interval was 2.1 years (interquartile range 1.5–3.0). Compared to pregnant women in the general population without bariatric surgery history, women with such history were older and were more likely to be obese, to smoke, to be of Nordic origin, and to be multiparous (all *P <* 0.001; Table 1). These distributional differences were eliminated by the matching procedure (Table 1).

**Table 1:** Maternal characteristics in singleton live term births in Sweden between 2006 and 2013.

Characteristic	Before matching		*P* value	After matching		*P* value^c^
	Births after bariatric surgery^a^	General population births^b^		Births after bariatric surgery^a^	Matched control births	
***N***	1,929	798,338		1,431	4,476	—
**Maternal age—years (mean ± SD)**	31 ± 5	30 ± 5	<0.001	31 ± 5	31 ± 5	<0.001^d^
**Surgery-to-conception interval**						
<1 year	675 (35.0%)	—	—	515 (36.0%)	—	1.0
1 to <2 years	644 (33.4%)	—		479 (33.5%)	—	
2 to <5 years	593 (30.7%)	—		428 (29.9%)	—	
≥5 years	17 (0.9%)	—		9 (0.6%)	—	
**Surgery-to-delivery interval**						
<1 year	83 (4.3%)			64 (4.5%)		
1 to <2 years	784 (40.6%)			598 (41.8%)		
2 to <5 years	1,013 (52.5%)			740 (51.7%)		
≥5 years	49 (2.5%)			29 (2.0%)		
**Maternal height—cm (mean ± SD)**	167 ± 6	166 ± 6	<0.001	167 ± 6	167 ± 6	0.007^d^
**BMI before surgery/matching—kg/m**^**2**^ **(mean ± SD)**	44.1 ± 5.7	—	—	42.6 ± 4.7	40.3 ± 4.2	<0.001^d^
30 to <35	37 (1.9%)	—	—	37 (2.6%)	174 (3.9%)	1.0
35 to <40	416 (21.6%)	—		382 (26.7%)	1,734 (38.7%)	
40 to <45	737 (38.2%)	—		648 (45.3%)	2,090 (46.7%)	
45 to <50	450 (23.3%)	—		286 (20.0%)	388 (8.7%)	
≥50	289 (15.0%)	—		78 (5.5%)	90 (2.0%)	
**Early-pregnancy BMI—kg/m**^**2**^ **(mean ± SD)**	30.0 ± 5.1	24.6 ± 4.6	<0.001	29.0 ± 4.5	40.3 ± 4.2	<0.001
<18.5	1 (0.1%)	17,885 (0.2%)	<0.001	1 (0.1%)	0	1.0
18.5 to <25	277 (14.4%)	449,247 (56.3%)		258 (18.0%)	0	
25 to <30	738 (38.3%)	185,670 (23.3%)		622 (43.5%)	0	
30 to <35	518 (26.9%)	62,414 (7.8%)		365 (25.5%)	174 (3.9%)	
35 to <40	215 (11.1%)	19,111 (2.4%)		122 (8.5%)	1,734 (38.7%)	
≥40	77 (4.0%)	6,877 (0.9%)		29 (2.0%)	2,568 (57.4%)	
*Missing*	103 (5.3%)	57,134 (7.2%)		34 (2.4%)	0	
**Smoking status**						
Non-smoker	1,594 (82.6%)	718,332 (90.0%)	<0.001	1,290 (90.1%)	4,138 (92.4%)	1.0
1–9 cigarettes/day	192 (10.0%)	38,097 (4.8%)		105 (7.3%)	276 (6.2%)	
≥10 cigarettes/day	71 (3.7%)	10,890 (1.4%)		29 (2.0%)	53 (1.2%)	
*Missing*	72 (3.7%)	31,019 (3.9%)		7 (0.5%)	9 (0.2%)	
**Educational level**						
<10 years	304 (15.8%)	77,900 (9.8%)	<0.001	184 (12.9%)	496 (11.1%)	1.0
10–12 years	1,163 (60.3%)	290,332 (36.4%)		889 (62.1%)	2,779 (62.1%)	
>12 years	460 (23.8%)	420,038 (52.6%)		357 (24.9%)	1,199 (26.8%)	
*Missing*	2 (0.1%)	10,068 (1.3%)		1 (0.1%)	2 (0.0%)	
**Primiparous**	785 (41%)	353,003 (44%)	<0.001	544 (38%)	1,664 (37%)	1.0
**Nordic country of birth**	1,705 (88%)	626,230 (78%)	<0.001	1,291 (90%)	4,048 (90%)	1.0

Values are number (percent) unless otherwise indicated.

^a^With data on pre-surgery weight available from the Scandinavian Obesity Surgery Registry.

^b^General population births without bariatric surgery history.

^c^P for continuous variables from 2-way anova, for categorical variables from logistic regression (both conditioned on the matching set).

^d^The mean between-group difference in age (mean difference 54 days, 95% CI 26–81), maternal height (mean difference 0.1 cm, 95% CI 0–0.2), and BMI (mean difference 0.64 kg/m^2^, 95% CI 0.55–0.73) was conditioned on the matching set. The small differences are caused by the fact that the matching was done by categories and that not all matching sets include a full 5 control births but may have 1, 2, 3, 4, or 5 matched controls.

Women with bariatric surgery history did not differ from matched controls regarding pre-surgery obesity status or age distribution, but still had a higher mean pre-surgery BMI (mean difference 0.64 kg/m^2^; 95% CI 0.55–0.73) and were on average 54 days older (95% CI 26–81; Table 1). In the surgery group, the mean BMI loss between surgery and early pregnancy was 13.5 kg/m^2^ (SD 4.2), corresponding to a weight loss of 38 kg (SD 12), or 31.7% (SD 8.5%) (Table 1; S2 Fig).

**Fig 1:**
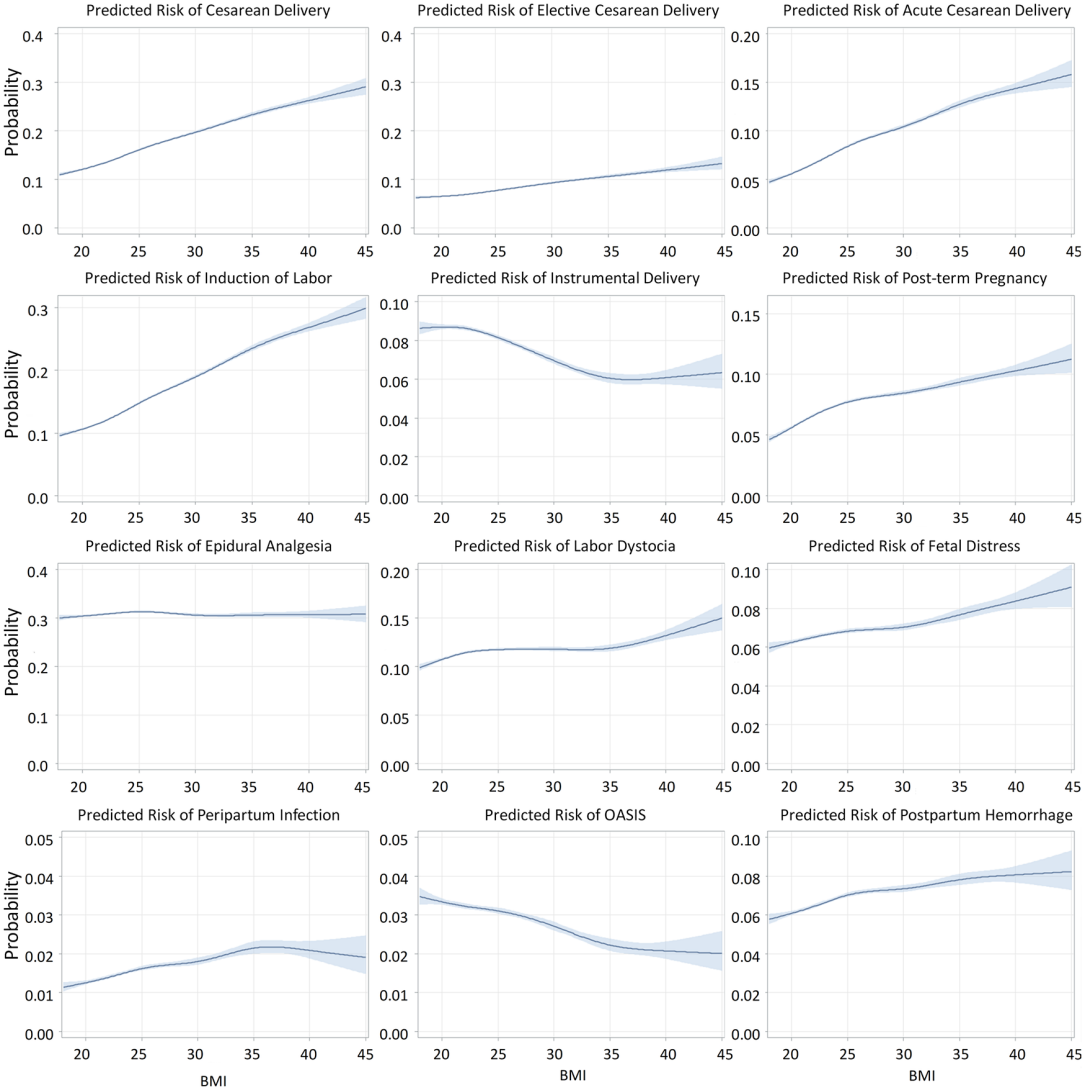
Relation between early-pregnancy BMI and delivery outcomes (*N =* 729,867* singleton live births at or after 37 gestational weeks). *X* axis is BMI (kg/m^2^); different *Y* axis scales. OASIS = obstetric anal sphincter injury (perineal tear grade III–IV). Acute (emergency) cesarean delivery = all unplanned cesarean sections. *798,338 minus 57,134 with missing BMI (excluding births to women with bariatric surgery history).

In Fig 1 we present the relation between maternal early-pregnancy BMI and adverse delivery outcomes based on 729,867 deliveries in women without bariatric surgery history. There was an association between increased BMI and the risk of cesarean delivery, induction of labor, post-term pregnancy, labor dystocia, fetal distress, postpartum hemorrhage, and peripartum infection. There was no association between BMI and epidural analgesia, and a negative association between BMI and OASIS.

**Fig 2:**
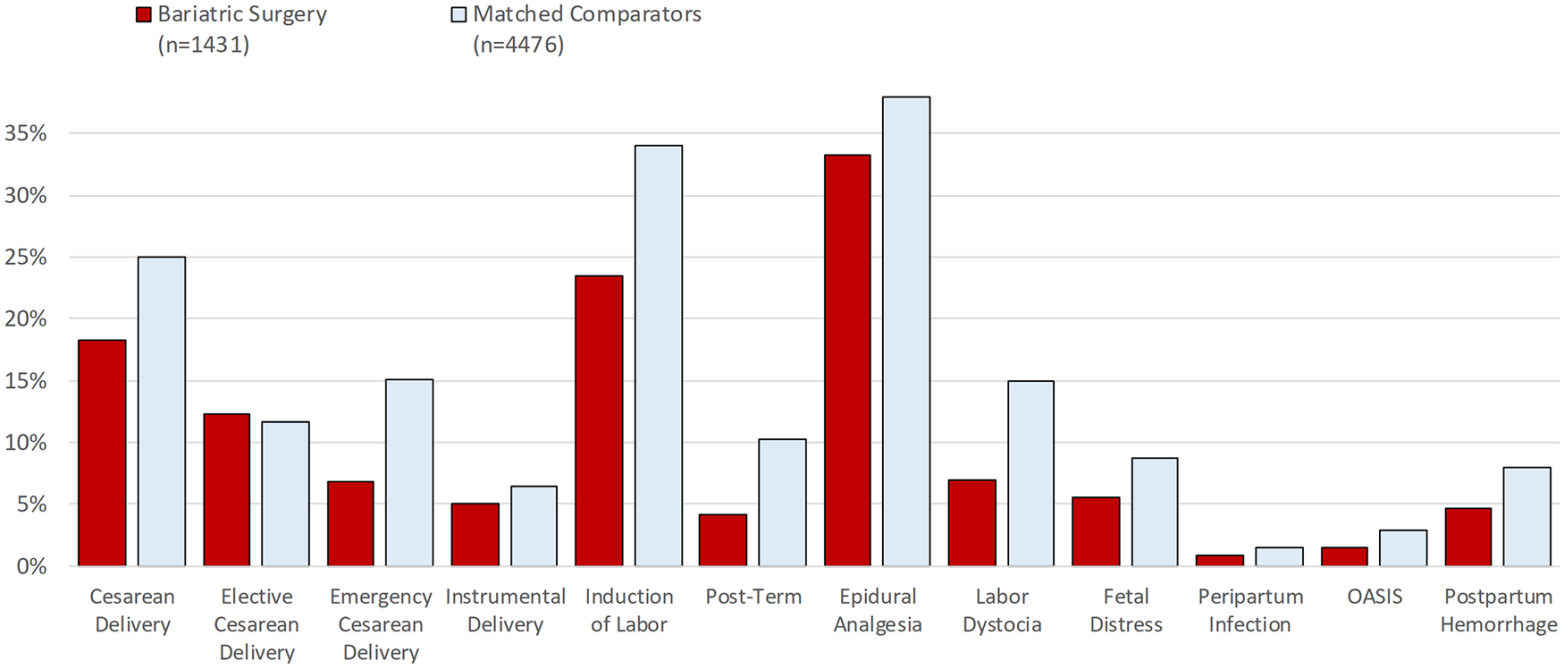
Delivery outcomes after bariatric surgery and in matched general population controls*. Elective cesarean deliveries were excluded in the analysis of emergency cesarean delivery, instrumental delivery, induction of labor, epidural analgesia, labor dystocia, and fetal distress (bariatric surgery: *N =* 1,255; matched comparators: *N =* 3,952). Cesarean deliveries were excluded in the analysis of OASIS (bariatric surgery: *N =* 1,170; matched comparators: *N =* 3,355). OASIS = obstetric anal sphincter injury (perineal tear grade III–IV). Emergency cesarean delivery = all unplanned cesarean sections. *Matching factors: maternal age, parity, pre-surgery BMI category (using early-pregnancy BMI in controls), early-pregnancy smoking status, educational level, height, country of birth, and delivery year.

**Fig 3:**
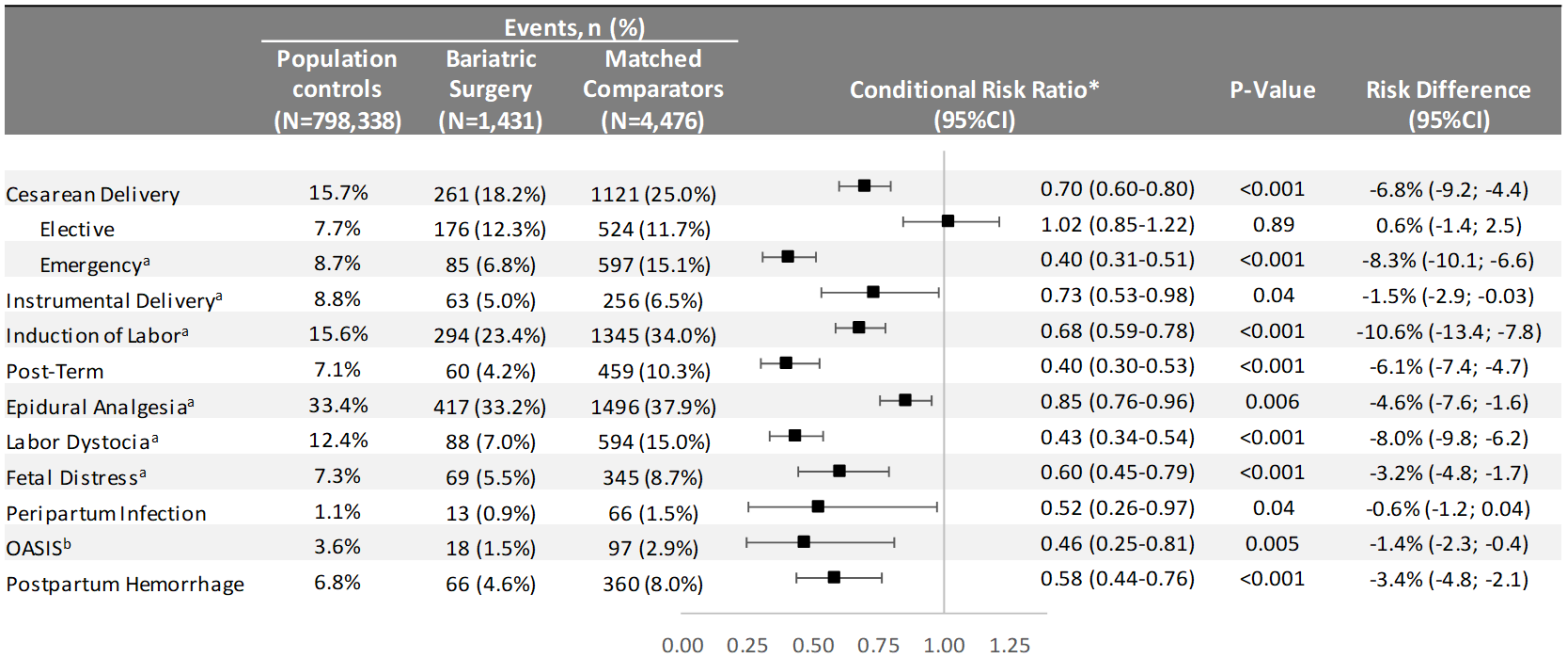
Delivery outcomes in singleton live term births in Sweden between 2006 and 2013. OASIS = obstetric anal sphincter injury (perineal tear grade III–IV). Emergency cesarean delivery = all unplanned cesarean sections. ^a^Elective cesarean deliveries excluded. ^b^Cesarean deliveries excluded. *Conditioned on the matching factors maternal age, parity, pre-surgery BMI category (using early-pregnancy BMI in controls), early-pregnancy smoking status, educational level, height, country of birth, and delivery year.

Absolute risks for delivery outcomes in women with bariatric surgery history and matched controls are presented in Fig 2 (S3 Fig also includes unmatched population comparators), and adjusted relative risks in Fig 3. Compared to matched population controls, post-surgery women were less likely to have cesarean delivery (18.2% versus 25.0%; RR 0.70, 95% CI 0.60–0.80), with the decreased risk observed for emergency cesarean delivery (6.8% versus 15.1%; RR 0.40, 95% CI 0.31–0.51), but not for elective cesarean delivery (12.3% versus 11.7%; RR 1.02, 95% CI 0.85–1.22). When analyzing by indication for emergency cesarean delivery, there was a lower risk of cesarean delivery with labor dystocia and fetal distress indication. The risk of instrumental delivery was 5.0% versus 6.5% in post-surgery women compared to matched controls (RR 0.73, 95% CI 0.53–0.98). Post-surgery women had lower risks of induction of labor, post-term pregnancy, and epidural analgesia. Risks were also reduced for labor dystocia, fetal distress, peripartum infection, OASIS, and postpartum hemorrhage.

### Subgroup analyses

**Fig 4:**
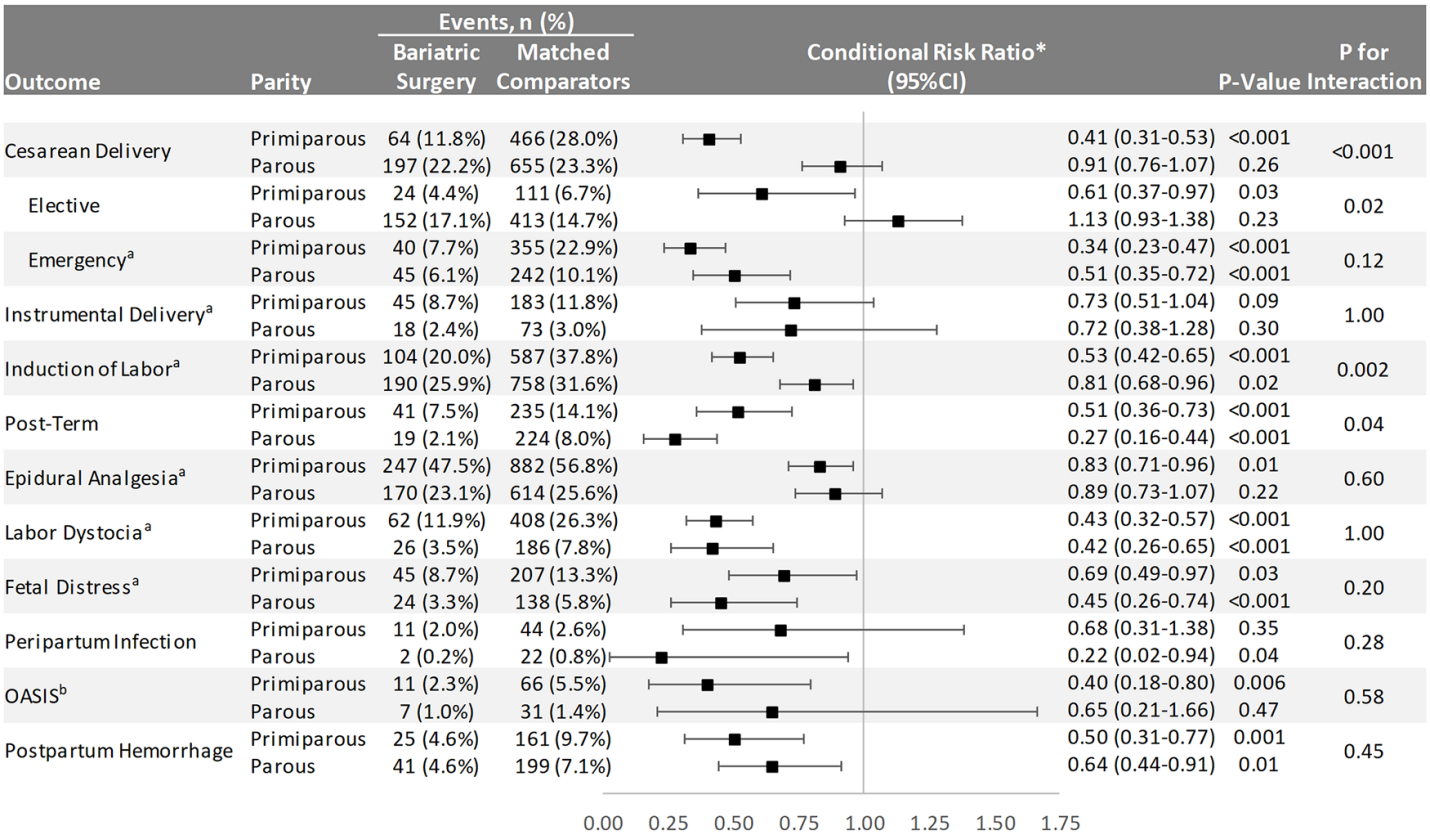
Delivery outcomes in singleton live term births in Sweden between 2006 and 2013 stratified by parity. OASIS = obstetric anal sphincter injury (perineal tear grade III–IV). Emergency cesarean delivery = all unplanned cesarean sections. ^a^Elective cesarean deliveries excluded. ^b^Cesarean deliveries excluded. *Conditioned on the matching factors maternal age, parity, pre-surgery BMI category (using early-pregnancy BMI in controls), early-pregnancy smoking status, educational level, height, country of birth, and delivery year.

Effect modification by parity was found for cesarean delivery (primarily driven by differences in elective cesarean sections; *P* = 0.02), with no difference versus controls in parous women (Fig 4). Statistically significant effect modification was also found for induction of labor (greater risk reduction in primiparous than parous women; *P* = 0.002) and post-term pregnancy (greater risk reduction in parous than primiparous women; *P* = 0.04; Fig 4).

**Fig 5:**
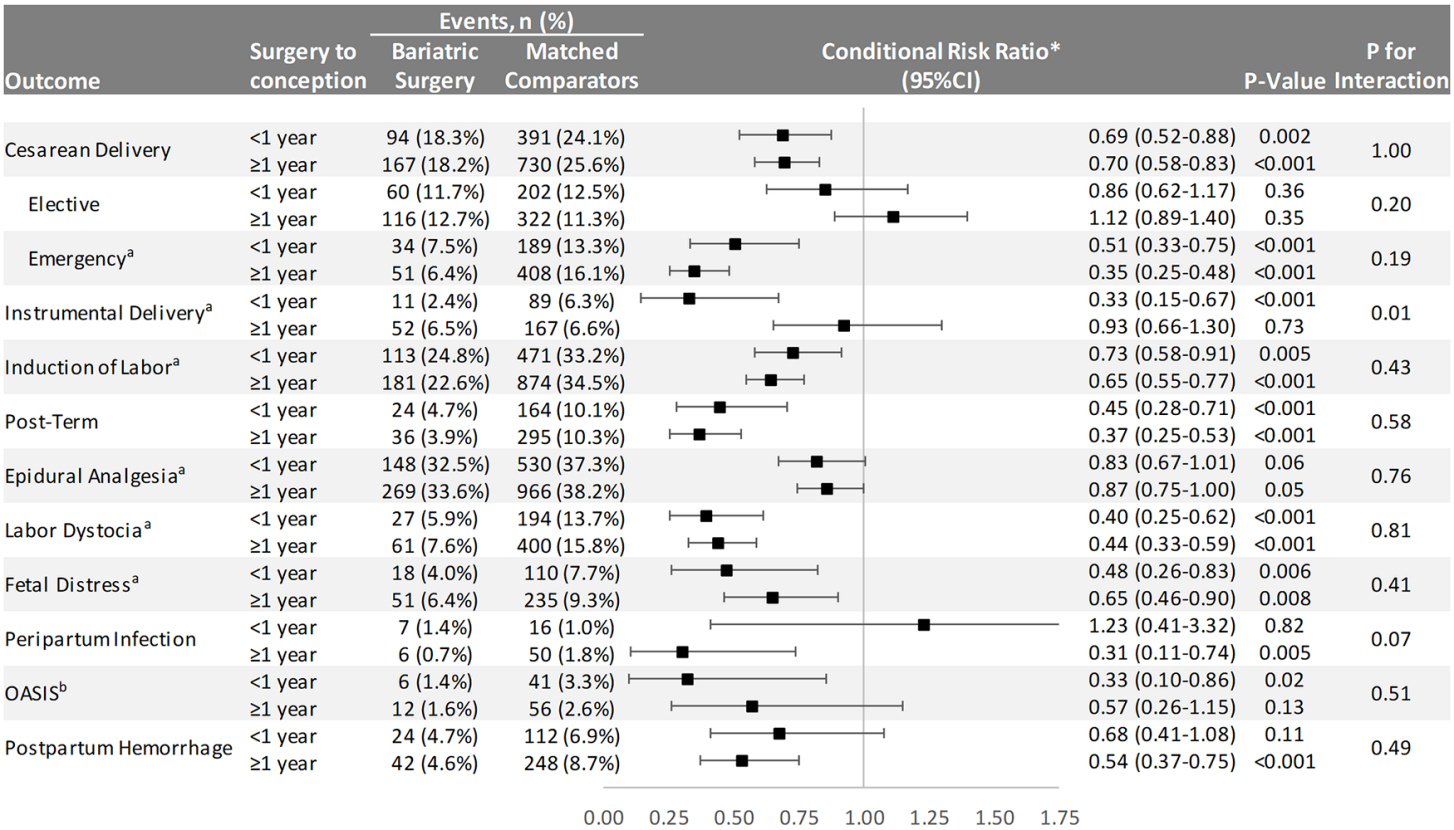
Delivery outcomes in singleton live term births in Swedish women between 2006 and 2013 stratified by surgery-to-conception interval. OASIS = obstetric anal sphincter injury (perineal tear grade III–IV). Emergency cesarean delivery = all unplanned cesarean sections. ^a^Elective cesarean deliveries excluded. ^b^Cesarean deliveries excluded. *Conditioned on the matching factors maternal age, parity, pre-surgery BMI category (using early-pregnancy BMI in controls), early-pregnancy smoking status, educational level, height, country of birth, and delivery year.

Regarding variations across surgery-to-conception interval categories, we found statistically significant effect modification for instrumental delivery, with greater protective effect for women who became pregnant during the first year after surgery (Fig 5).

### Sensitivity analysis

In the main analysis, we did not account for clustering due to women giving birth more than once after bariatric surgery (*N =* 142; 9.9%). Therefore, we also performed a sensitivity analysis including only the first birth after surgery, resulting in similar estimates as in the main analysis (S1 Table).

### Mediation analysis

In a mediation analysis where we adjusted for all matching variables and birth weight (mean birth weight in women with history of bariatric surgery was 3,458 g, and in the matched controls 3,813 g; *P <* 0.001), only minimal attenuation in results was observed as compared to the main model (S2 Table).

## Discussion

### Main findings

In this nationwide matched prospective cohort study, women with bariatric surgery history had lower risk of instrumental delivery and cesarean delivery during labor than controls matched on pre-surgery BMI. Furthermore, the risks of post-term pregnancy, induction of labor, epidural analgesia, labor dystocia, fetal distress, peripartum infection, OASIS, and postpartum hemorrhage were substantially lower in women with bariatric surgery history compared with the matched controls. In analyses stratified by parity, the reduced risk for cesarean delivery and OASIS was restricted to primiparous women. Furthermore, the reduced risk for instrumental delivery was only observed in women with <1 year between surgery and conception.

### Previous research

Reduction in cesarean deliveries was most pronounced for emergency cesareans, whereas the proportion of elective cesareans did not differ between women with bariatric surgery history and BMI-matched controls. Lesko and Peaceman [17] studied the risk of cesarean section in 70 women after bariatric surgery as compared to 140 controls matched on pre-surgery BMI, but did not report a lower risk. A US study by Abenhaim et al. using controls with class III obesity (BMI ≥ 40 kg/m^2^) found a lower risk of cesarean delivery overall [23], whereas other studies have not reported lower risk [6,24,25]. When we investigated indication for emergency cesarean, we found strong associations of emergency cesarean with labor dystocia and fetal distress, which no previous study to our knowledge has investigated. The lower risk estimates for labor induction in post-surgery deliveries observed in our study are in contrast with the findings of Abenhaim et al. [23] and an Israeli study of 326 cases compared with obese controls, which reported increased risk of labor induction in post-surgery deliveries [22]. A lower risk of post-term pregnancy but not labor induction was reported in a Danish cohort study with controls matched by early-pregnancy (rather than pre-surgery) BMI [6]. Rates of labor induction in the present study were lower in post-surgery deliveries. This could partially be attributed to a lower proportion of post-term pregnancy, which is a major indication for labor induction.

The almost halved risk of postpartum hemorrhage in post-surgery women is important, given that postpartum hemorrhage is a major cause of maternal morbidity and also mortality, and is supported by previous findings in studies that included obese controls [23,24] and controls matched on pre-surgery BMI [17].

Although BMI was negatively associated with OASIS risk, we observed a decreased risk for OASIS in women with bariatric surgery history compared with matched controls. OASIS is associated with incontinence and reduction in quality of life and also has major implications for reproductive health [32]. This is a novel finding, and could be seen despite the negative correlation of OASIS risk with maternal BMI [33].

Previous studies have reported a higher proportion of small-for-gestational-age infants and lower proportion of large-for-gestational-age infants born to mothers with bariatric surgery history [9,17,23], suggesting a shift towards lower risk of excessive fetal growth. The lower birth weight is also influenced by a lower proportion of post-term pregnancy, which is a major cause for macrosomia.

### Mechanisms

We hypothesized that the probable mechanism behind a lower risk of adverse delivery outcomes was the reduction in fetal size at birth. However, in the mediation analysis, introduction of birth weight did not have a major impact on the reduced risk of delivery outcomes observed. Hence, bariatric-surgery-induced weight loss appears to have beneficial effects on delivery outcomes independent of reduction in fetal growth. The reduction in delivery complications in mothers with bariatric surgery such as postpartum hemorrhage, OASIS, and peripartum infection may be caused by shorter duration of first and second stage labor, with fewer interventions and examinations and other factors associated with severe obesity in the mother. Furthermore, reduction in birth weight also decreases risk of uterine atony as well as birth canal laceration. We have previously reported a lower risk of gestational diabetes after bariatric surgery, which could lower the risk of labor induction, and also lower birth weight compared to matched control women [9]. Although there was no association between increased BMI and instrumental delivery, we found a reduced risk of instrumental delivery in women with previous bariatric surgery compared to matched population controls, which was unexpected.

### Strengths and limitations

Our study was of sufficient size to detect clinically meaningful differences in delivery outcomes between women with bariatric surgery history and matched controls. Further, for the women with bariatric surgery history, we had access to and matched on pre-surgery BMI instead of early-pregnancy BMI, which is often used but addresses a different research question. Pre-surgery matching answers the question of the effect of bariatric surgery itself, including weight loss and other metabolic/anatomical effects, on outcomes during pregnancy and delivery, while early-pregnancy BMI matching corresponds to a more clinical question, i.e., whether 2 women with similar early-pregnancy BMI, but one with a history of bariatric surgery and the other without such history, are expected to have similar outcomes during pregnancy [6,21,34].

Some studies have compared women with previous bariatric surgery to obese women or morbidly (class III) obese women in early pregnancy [20,22–24]. Such an approach is less precise than comparing based on pre-surgery BMI, which in our study ranged between 30 and 70 kg/m^2^. In contrast to the studies by Abenhaim et al. [23] and Parker et al. [24], which were based on delivery hospital discharge codes for pregnancies complicated by previous bariatric surgery, we used prospective exposure information from the high-quality SOReg, which covers 98% of all bariatric surgery procedures in Sweden (data are entered into the register by the surgeon who performed the bariatric surgery procedure). This approach is a major strength of our study and ensures that misclassification of the exposure is very limited in our study as compared to studies relying on discharge codes.

The study is based on data from the Swedish national health registers linked with SOReg. These health registers have a high quality, although there may be a proportion of up to 5% missing data for some variables [26,28,29].

This study was not randomized: Randomisation requiring pregnancy after bariatric surgery and a control intervention would be impossible to implement. The observational design may be affected by selection bias, despite our efforts using careful matching. Another potential limitation is that we used pre-surgery data regarding maternal BMI for post-surgery births, but data from early pregnancy for matched controls. Given the median surgery-to-conception interval of 1.4 years, we believe this was a reasonable approach.

Despite matching by BMI category, women with bariatric surgery history had significantly higher BMI at matching than controls. This is likely to result in a conservative bias, as greater BMI is associated with increased risks for most of the investigated outcomes. In the matching procedure, 26% of post-surgery pregnancies were excluded, foremost in the highest BMI category (BMI ≥ 50 kg/m^2^), because no matched controls could be identified. We were able to take into account the influence of country of birth but did not have access to information on ethnicity, which is a limitation affecting generalisability.

The vast majority of bariatric surgery procedures were gastric bypass, a procedure with greater malabsorptive effects than, for example, sleeve gastrectomy or gastric banding. Hence, our results may not be generalisable to women with other procedures.

A limitation was that we did not have data on indication for induction of labor. Furthermore, dystocia was based on diagnoses at discharge from the delivery hospital and was therefore more prevalent in women with instrumental or cesarean delivery as compared to non-instrumental vaginal delivery. However, we do not think that dystocia diagnosis was influenced by bariatric surgery history, and any misclassification would therefore be non-differential.

### Conclusion

Bariatric-surgery-induced weight loss was associated with lower risk of cesarean and instrumental delivery and substantially lower risks of maternal complications during delivery and the early postpartum period. Given the magnitude of the observed effects, bariatric surgery may be an important procedure for improving delivery outcomes in obese and morbidly obese women. However, there is also an increased risk of complications for the infant, including small-for-gestational-age and preterm birth, that has to be taken into consideration.

## Supporting information

S1 Fig Flow chart showing the identification of births after bariatric surgery and their matched control births. (DOCX)

S2 Fig Smoothed curves of early-pregnancy BMI and pre-surgery BMI in women giving birth after bariatric surgery. (DOCX)

S3 Fig Delivery outcomes in the general population, after bariatric surgery, and in matched general population controls. (DOCX)

S1 Table Mediation analysis including birth weight (continuous variable) in delivery outcomes. (DOCX)

S2 Table Delivery outcomes of the first singleton live term birth in women in Sweden between 2006 and 2013. (DOCX)

## Abbreviations

BMI: body mass index
OASIS: obstetric anal sphincter injury
RR: risk ratio
SOReg: Scandinavian Obesity Surgery Registry
