# Supplementary material for: Delivery outcomes in term births after bariatric surgery: Population-based matched cohort study
Source: PLoS Med. 2018 Sep 26;15(9):e1002656. doi: 10.1371/journal.pmed.1002656 (PMC6157842; doi:10.1371/journal.pmed.1002656)
Supplement: S2 Fig — (DOCX) [file pmed.1002656.s002.docx]

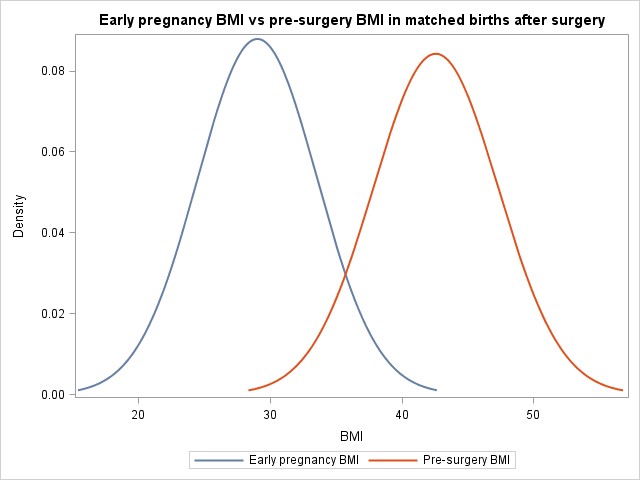


**S2 Fig** Smoothed curves of early pregnancy BMI and pre-surgery BMI in women giving birth after bariatric surgery
