# Supplementary material for: Delivery outcomes in term births after bariatric surgery: Population-based matched cohort study
Source: PLoS Med. 2018 Sep 26;15(9):e1002656. doi: 10.1371/journal.pmed.1002656 (PMC6157842; doi:10.1371/journal.pmed.1002656)
Supplement: S3 Fig — (DOCX) [file pmed.1002656.s003.docx]

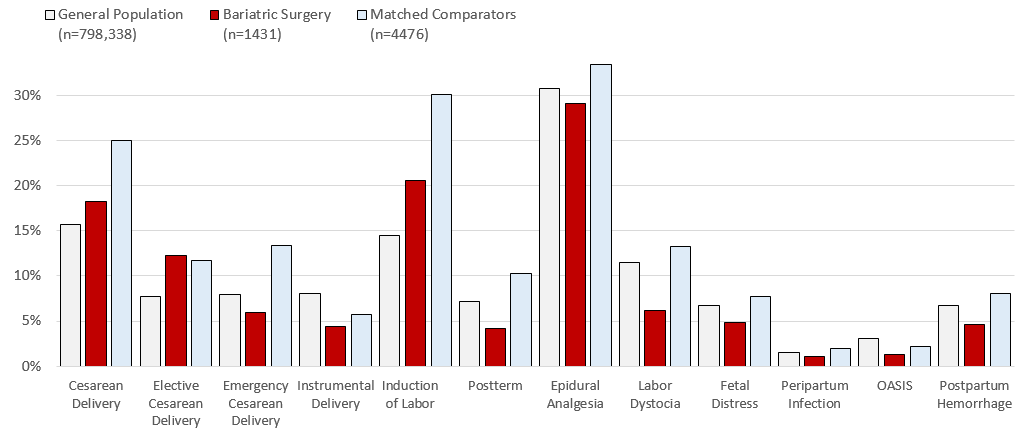


**S3 Fig** Delivery outcomes in the general population, after bariatric surgery, and in matched general population controls^[[1]](#footnote-2)^

1. Matching factors: Maternal age, parity, pre-surgery BMI-category (using early-pregnancy BMI in controls), pregnancy smoking status, educational level, height, country of birth, and delivery year [↑](#footnote-ref-2)
