# Supplementary material for: Delivery outcomes in term births after bariatric surgery: Population-based matched cohort study
Source: PLoS Med. 2018 Sep 26;15(9):e1002656. doi: 10.1371/journal.pmed.1002656 (PMC6157842; doi:10.1371/journal.pmed.1002656)
Supplement: S2 Table — (DOCX) [file pmed.1002656.s005.docx]

**S2 Table** Maternal delivery outcomes of the first singleton live term births in women in Sweden between 2006 and 2013

| **Restriction: Only First Birth After Surgery Included** | **N** | | **Events, n (%)** | | **Conditional  Risk Difference**  **(95%CI)** | **Conditional**  **Risk Ratio***  **(95%CI)** | **P** |
| --- | --- | --- | --- | --- | --- | --- | --- |
|  | **Bariatric  Surgery** | **Matched Comparators** | **Bariatric Surgery** | **Matched**  **Comparators** |  |  |  |
| Caesarean Delivery | 1289 | 4057 | 235 (18.2%) | 1011 (24.9%) | -6.7% (-9.2; -4.2) | 0.70 (0.60-0.81) | <0.001 |
| Elective | 1289 | 4057 | 156 (12.1%) | 451 (11.1%) | 1.0% (-1.0; 3.0) | 1.05 (0.86-1.27) | 0.67 |
| Emergency | 1133 | 3606 | 79 (7.0%) | 560 (15.5%) | -8.6% (-10.5; -6.7) | 0.40 (0.31-0.52) | <0.001 |
| Instrumental Delivery | 1133 | 3606 | 57 (5.0%) | 243 (6.7%) | -1.7% (-3.2; -0.2) | 0.70 (0.50-0.95) | 0.02 |
| Induction of Labour | 1133 | 3606 | 263 (23.2%) | 1234 (34.2%) | -11.0% (-13.9; -8.1) | 0.67 (0.58-0.78) | <0.001 |
| Postterm | 1289 | 4057 | 52 (4.0%) | 424 (10.5%) | -6.4% (-7.8; -5.0) | 0.38 (0.28-0.51) | <0.001 |
| Epidural Analgesia | 1133 | 3606 | 391 (34.5%) | 1410 (39.1%) | -4.6% (-7.8; -1.4) | 0.84 (0.74-0.95) | 0.004 |
| Labour Dystocia | 1133 | 3606 | 81 (7.1%) | 561 (15.6%) | -8.4% (-10.3; -6.5) | 0.42 (0.33-0.54) | <0.001 |
| Fetal Distress | 1133 | 3606 | 64 (5.6%) | 323 (9.0%) | -3.3% (-4.9; -1.7) | 0.60 (0.45-0.80) | <0.001 |
| Peripartum Infection | 1289 | 4057 | 13 (1.0%) | 64 (1.6%) | -0.6% (-1.2; 0.1) | 0.53 (0.26-1.00) | 0.05 |
| OASIS | 1054 | 3046 | 18 (1.7%) | 93 (3.1%) | -1.3% (-2.3; -0.4) | 0.48 (0.26-0.83) | 0.007 |
| Postpartum Haemorrhage | 1289 | 4057 | 60 (4.7%) | 321 (7.9%) | -3.3% (-4.7; -1.8) | 0.60 (0.44-0.80) | <0.001 |

*Conditioned on the matching factors: maternal age, parity, pre-surgery BMI-category (using early-pregnancy BMI in controls), early-pregnancy smoking status, educational level, height, country of birth, and delivery year. OASIS = obstetric anal sphincter injury (perineal tear grade III-IV). Emergency caesarean delivery = all unplanned caesarean sections.
